# Signal management and risk minimization strategy: a case study on obinutuzumab and non-overt disseminated intravascular coagulation

**DOI:** 10.3389/fdsfr.2023.1194683

**Published:** 2023-07-17

**Authors:** Giulia Valdiserra, Nadia Mores, Rosalba Elisabetta Rocchi, Laura Sottosanti, Patrizia Felicetti, Pasquale Marchione, Luca Laurenti, Alberto Fresa, Giampaolo Bucaneve, Emiliano Cappello, Marco Bonaso, Sara Ferraro, Irma Convertino, Marco Tuccori

**Affiliations:** ^1^ Unit of Pharmacology and Pharmacovigilance, Department of Clinical and Experimental Medicine, University of Pisa, Pisa, Italy; ^2^ Department of Pharmacology, Faculty of Medicine, Catholic University of the Sacred Heart, University Hospital Agostino Gemelli Foundation, Rome, Italy; ^3^ Centro Regionale Farmacovigilanza—Regione Umbria, Perugia, Italy; ^4^ Agenzia Italiana del Farmaco, Rome, Italy; ^5^ Department of Hematology, Faculty of Medicine, Catholic University of the Sacred Heart, University Hospital Agostino Gemelli Foundation, Rome, Italy; ^6^ Unit of Adverse Drug Reactions Monitoring, Pisa University Hospital, Centro Regionale Farmacovigilanza Toscana, Pisa, Italy

**Keywords:** obinutuzumab, disseminated intravascular coagulation, signal detection, risk minimization, signal validation, signal confirmation, signal analysis and prioritization, signal assessment

## Abstract

**Introduction:** In December 2020, the Italian Medicines Agency (AIFA) in collaboration with the Italian Regional Centers of Pharmacovigilance evaluated four individual case safety reports (ICSRs) reporting obinutuzumab and non-overt disseminated intravascular coagulation (DIC) as a new possible signal. In this study, we described the process of signal management for obinutuzumab-associated non-overt DIC.

**Methods:** In accordance with the Guideline on Good Pharmacovigilance Practices Module IX, we described the process of the Italian and European Union signal management process in five steps: signal detection, signal validation, signal confirmation, signal analysis, and prioritization and signal assessment.

**Results:** In the signal detection phase, four cases of obinutuzumab-associated non-overt DIC met the criteria for signal definition (ROR 213.6 and IC025 77). In the signal validation phase, both the Italian and European databases of spontaneously reported adverse drug reactions were investigated with supporting evidence from medical literature. Four patients (two men and two women aged 67–77) were in treatment with obinutuzumab for chronic lymphocytic leukemia, and they developed a subclinical DIC within 24 h from the administration of the drug. The DIC spontaneously resolved in all cases. Three more ICSRs were reported in the EudraVigilance database. The medical literature provided poor evidence of the possible association between obinutuzumab and DIC. The signal was subsequently validated, first by AIFA and then by EMA. The signal was confirmed by the Pharmacovigilance Risk Assessment Committee (PRAC) Rapporteur in the “signal confirmation” phase, and it entered “signal analysis and prioritization” and “signal assessment”. In these phases, the PRAC assessed and confirmed DIC as a possible adverse reaction to obinutuzumab. Finally, the Summary of Product characteristics was updated with this new risk.

**Conclusion:** Despite the intrinsic difficulties linked to the nature of the event itself, the process of signal detection and the issuing of the risk minimization measures by the Italian Medicines Agency as part of the EU procedures have proven to be efficient.

## Introduction

Obinutuzumab is a glycoengineered, humanized, type II, monoclonal antibody (mAb) anti-CD20 of the immunoglobulin G1 subclass. It has been approved in the EU since 2014 (European Medicines Agency, 2022a) and in the USA since 2016 (U.S. Food and Drug Administration, 2022) for the treatment of adults with chronic lymphocytic leukemia (CLL) who were never treated or were not eligible for treatment with a full dose of fludarabine and chlorambucil. It is also indicated for the treatment of not-pretreated adults with advanced follicular lymphoma (disease progression within 6 months of treatment with rituximab) in combination with chemotherapy or with bendamustine. Adverse drug reactions (ADRs) for obinutuzumab include cough (20%), neutropenia (13%), upper respiratory tract infections (12%), diarrhea (10%), nausea (9%), infusion-related reactions (8%), and skin rash (6%). The most common grade 3–5 ADRs are neutropenia and febrile neutropenia, anemia, thrombocytopenia, and upper respiratory or urinary tract infections (Hoy, 2015).

Disseminated intravascular coagulation (DIC), also called consumption coagulopathy, is a coagulation disorder triggered by systemic inflammation resulting in a deposition of fibrin and platelet activation. Therefore, patients with DIC are at risk for both thrombosis and bleeding (Levi and Scully, 2018). DIC can often occur as a complication of disorders that can affect coagulation. The clinical conditions most frequently associated with DIC are infectious diseases, malignancy, trauma, obstetrical complications, vascular malformations, severe immunological reactions, heat stroke, and post-cardiopulmonary resuscitation (Levi, 2018). DIC also is described in the analysis of the World Health Organization VigiBase^®^ database as an adverse event for some drugs, including antineoplastic, antithrombotic agents, and antibacterials for systemic use. However, the Summary of Product Characteristics (SPC) has barely been updated with this ADR (Bonaldo et al., 2020).

In December 2020, during signal detection activities, the Italian Medicines Agency (AIFA), in collaboration with Italian Regional Pharmacovigilance (PV) Centers, identified a signal for a possible new association between obinutuzumab and non-overt DIC that was subsequently acknowledged as a possible ADR and was listed in the obinutuzumab SPC. This study describes the process of signal detection from identification to the final issuing of risk minimization measures.

## Materials and methods

### Data source

The Italian spontaneous reporting system, managed by AIFA, is called the National PV Network (RNF—Rete Nazionale di Farmacovigilanza). Since January 2001, RNF has collected individual case safety reports (ICSRs) sent by Italian healthcare professionals or citizens. The ADR reports are spontaneous or from non-interventional studies or special use (i.e., compassionate use, nominal therapeutic use, and Law 648/1996). They are coded using the Medical Dictionary for Regulatory Activities (MedDRA), while drugs are categorized according to the Anatomical Therapeutic and Chemical (ATC) Classification. RNF data are managed with the support of the Italian Regional Centers of Pharmacovigilance, which are responsible for data quality monitoring, including the collection of follow-up information. The RNF has been already used as an information source for signal detection activity by other authors (Crisafulli et al., 2022; Cutroneo et al., 2017; Capogrosso Sansone et al., 2017).

### Data analysis

The process of signal detection and management is conducted by AIFA in collaboration with the Italian Regional PV Centers in accordance with the Guideline on Good Pharmacovigilance Practices (GVP) Module IX (European Medicines Agency, 2017). The RNF is checked daily by AIFA and the Regional PV Centers for emerging new signals of drug- or vaccine-related risks, and results are presented and discussed in at least four standard plenary meetings every year (two dedicated to vaccines and two to all other medicinal products). Extraordinary meetings can be required in emergency situations or whenever an emerging signal deserving discussion is identified.

When a signal is qualified within an AIFA working group because it is considered robust enough, an assessment report is submitted to the Rapporteur country or Lead Member State (depending on the type of EU marketing authorization procedures) as a proposal for evaluation by the Pharmacovigilance Risk Assessment Committee (PRAC) at the European Medicine Agency. The signal management process is summarized in Table 1.

**TABLE 1:** Summary of the signal management process.

Steps involved in the signal management process	Guideline on Good Pharmacovigilance Practices (GVP) Module IX	Signal management of obinutuzumab and DIC
Signal detection	Process of looking for and/or identifying signals using data from any source	Screening of the RMR by AIFA and the Regional Pharmacovigilance Centers in which all drug–event pairs that met the definition of disproportion were selected. Among these pairs were obinutuzumab and DIC
Signal validation	Process of evaluating data supporting the detected signal to verify that the available documentation contains sufficient evidence demonstrating the existence of a new potentially causal association or a new aspect of a known association and therefore justifies analysis of the signal	AIFA and the Regional Pharmacovigilance Centers investigated case by case the causal relationship for the ICSRs in the Italian and European databases. The medical literature was screened too. The signal was validated and entered into EPITT
Signal confirmation	Process of deciding whether a validated signal entered in EPITT requires further analysis and prioritization by the PRAC. This should be carried out by the PRAC Rapporteur or the Lead Member State within 30 days of receipt of the validated signal	Signal confirmed by the PRAC Rapporteur (Sweden)
Signal analysis and prioritization	Process by which the PRAC determines whether a confirmed signal requires further assessment, and if so, in what timeframe and in which procedural framework. This is based on an initial analysis of the potential impact of the signal on patient or public health and the risk–benefit balance of the concerned medicinal product	The PRAC assessed and confirmed DIC as a possible adverse reaction to obinutuzumab, the majority of which were non-overt DIC
Signal assessment	Following PRAC initial signal analysis and prioritization, the process of evaluating all available data relevant to a signal to determine the need for any regulatory action. This is led by the Rapporteur appointed by the PRAC following analysis and prioritization.	In February 2022, risk minimization measures were taken:
		1-updating paragraph 4.8 of the SPC with the PT “disseminated intravascular coagulation” with “uncommon” frequency;
		2-including a special warning in paragraph 4.4 of the SPC on the management of patients undergoing obinutuzumab administration.

* AIFA, Agenzia Italiana del Farmaco; DIC, disseminated intravascular coagulation; EPITT, European Pharmacovigilance Issues Tracking Tool; PRAC, Pharmacovigilance Risk Assessment Committee; SPC, Summary of Product Characteristics; RMR, Reaction Monitoring Report.

### Signal detection

The first step of signal management is “signal detection.” This phase is based on the examination of a file named “Reaction Monitoring Report” (RMR) that lists all the drug–event pairs collected in the RNF database that are periodically created. The reference periods may vary from 1 to 6 months. The RMR file is organized in lines and columns to provide all the essential information for manual screening of drug–event pairs. For each index drug–event pair, the file reports the ATC classification and the name of the active substance for the drug, the Medical Dictionary for Regulatory Activities (MedDRA) coding by System Organ Class (SOC) and preferred term (PT), together with the eventual classification as an important medical event (IME) (European Medicines Agency, 2023a). In the RMR file, the number of ICSRs for the drug–event pair is also listed for both the reporting period and as a total from the entire database, and for both the index drug and all the other drugs as well as the number of ICSRs for all the events other than the index one—again, for both the index drug and all the other drugs but the index. The crude ROR for each drug–event pair together with the lower and the upper limits of the 95% confidence interval (CI) are also presented in the RMR. Furthermore, a column containing a flag for easy recognition of the disproportion (yes or no) is present. A red cell in the column is generated automatically for a single pair every time the criteria for disproportion are met: number of ICSR ≥ 3 and ROR 95% CI lower limit > 1 (European Medicines Agency, 2017).

The RMR file is circulated by AIFA among the Regional PV Centers. It is manually screened independently by several Regional PV Centers, grouped by assigned ATC and AIFA for the identification of all the drug–event pairs that meet the criteria for disproportion that are selected for further analysis, case by case. Priority is given to the pairs based on expectedness number (unexpected events) and seriousness (clinically relevant events). Based on these criteria, Regional PV Centers or AIFA may decide to propose for plenary discussion the identified drug–event pair of interest. A preliminary decision is taken during the discussion. A signal can be proposed for further investigation (identified signal) and consequently enter the next step of the process of signal management, or it can be archived (as a rejected signal). Archived signals are not definitively closed but are periodically re-evaluated every time new reports become available, thus increasing the robustness of the disproportion.

### Signal validation

The second step of the process is “signal validation.” In this phase, the Regional PV Centers and AIFA investigate case-by-case the causal relationship between drug and event for all ICSRs of interest, present in both the Italian and the EudraVigilance databases. ICSRs are analyzed to identify cases that provide high quality and complete information for causality assessment. The medical literature is also explored for clinical, pharmacological, and pharmacoepidemiological evidence that supports an association between exposure to the suspected drug and the occurrence of the index event. As reported on the GVP, “*If the investigations show that the available documentation contains sufficient evidence demonstrating the existence of a new potentially causal association*, *or a new aspect of a known association*, *justifies further analysis*, *the signal is validated*” (European Medicines Agency, 2017). Validated signals are finally endorsed by the Support and Coordination Secretariat Post Marketing Area (SSCAVPM—Segretariato di Supporto e Coordinamento dell’Area Vigilanza Post Marketing), a multidisciplinary commission with Italian experts that, in collaboration with the PV Office, performs preliminary investigations. At the European level, the regulatory authority that validated the signal should enter it in the EPITT tracking tool.

### Signal confirmation

The third step is “signal confirmation,” in which the PRAC Rapporteur or the Lead Member State (LMS) responsible for the index drug confirms the signal or not. The LMS is responsible for the examination of drugs authorized by national, mutually recognized, or decentralized procedures, while the PRAC Rapporteur is responsible for drugs authorized with centralized procedures. Once the PRAC Rapporteur or the LMS confirms it is moved further along the path and is proposed for evaluation by the PRAC.

### Signal analysis and prioritization and signal assessment

The next two steps are represented by the “signal analysis and prioritization” and the “signal assessment” run at the EMA level, where the PRAC decides, based on the potential impact of newly proposed safety information, on patient or public health, and on the risk–benefit balance of the index drug, and whether the confirmed signal deserves further investigation, thus defining the timing and type of procedural framework. The marketing authorization holder (MAH) can also contribute to the assessment of the signal by providing additional data for assessment within the signal procedure or in the PSUSA procedure. Signal management ends with recommendations on signals by the PRAC that may induce one or a combination of conclusions, such as:

• No need for further evaluation or action at present;
• Need for additional information;
• Need for regulatory action—product information and/or risk management plan should be updated through a variation.

The risk minimization measures are communicated directly to the MAH and published on the EMA website. PRAC recommendations for regulatory action such as variation are submitted to the Committee for Medicinal Products for Human Use (CHMP) for endorsement when centrally authorized medicinal products are involved, and to the Co-ordination Group for Mutual Recognition and Decentralized Procedures—Human (CMDh) for information in the case of nationally authorized medicinal products. Each single national competent authority of Member States should then take appropriate measures (European Medicines Agency, 2023b).

## Results

In the signal detection phase on 9 December 2020, the obinutuzumab and disseminated intravascular coagulation pair met the criteria for signal definition (four reports, ROR: 213.6 and lower limit of the 95% CI of the ROR: 77) and priority for expectedness (not labeled) and seriousness (important medical event/life-threatening condition).

In the signal identification phase, the Regional PV Centers of Tuscany, Lazio, and Umbria analyzed the four ICSRs in the RNF together with AIFA. All the ICSRs were reported by a hematologist of the University Hospital Agostino Gemelli Foundation in Rome. The patients were two men and two women with a mean age of 74 years, treated with obinutuzumab and chlorambucil for CLL. They had concomitant therapies (i.e., anticoagulants, antihypertensives, protonic pump inhibitors, antibiotics, and angiotensin receptor blockers) for mostly age-related comorbidities. Concomitant drugs and comorbidities did not constitute risk factors for developing DIC. The CLL can be a risk factor for DIC but usually, in this clinical setting, the activation of coagulation is protracted. Furthermore, in some cases, the first clinical symptom may be hemorrhage in the tumor site. In all cases, a non-overt DIC was diagnosed in three patients 1 day after the administration of obinutuzumab and in one patient on the same day. Non-overt DIC is characterized by the absence of clinical symptoms—it was diagnosed on the basis of the differences in the results of scheduled hematological exams, performed before and after obinutuzumab administration (Fresa et al., 2021). The coagulation parameters were altered, showing in detail an increased D-dimer level, prolonged prothrombin time (PT), reduction of platelet counts, antithrombin III (AT III), and fibrinogen (see Table 2). An algorithm developed by the International Society on Thrombosis and Hemostasis (ISTH) was used for diagnosing DIC (Toh and Hoots, 2007)^,^(Taylor Jr et al., 2001). Non-overt DIC had a spontaneous resolution in all cases, and there was no need to discontinue the treatment with obinutuzumab. All patients completed the expected six cycles of treatment without any relapse of DIC. The causality assessment by the Naranjo scale was “possible” in each case.

**TABLE 2:** Summary of coagulation parameters of patients.

	Coagulation parameter	Day of infusion	Day of the ADR	6 days after the infusion
		12/01/2018 (C1D1)	13/01/2018 (C1D2)	19/01/2018 (C1D8)
Patient 1	INR	1.02	1.12	1.05
	PT	11.2 s	12.2 s	11.5 s
	aPTT	30.8 s	33.5 s	30.4 s
	Fibrinogen	352 mg/dL	310 mg/dL	239 mg/dL
	D-Dimer	1132 ng/mL	34687 ng/mL	499 ng/mL
	Antithrombin	88%	75%	93%
	Platelet	274000/mmc	92000/mmc	253000/mmc

	Coagulation parameter	Day of infusion	Day of the ADR	3 days after the infusion
		19/11/2019 (C1D1)	20/11/2019 (C1D2)	23/11/2019 (C1D5)
Patient 2	INR	2.87	3.61	1.03
	PT	27.9 s	34.4 s	10.8 s
	aPTT	48.5 s	59.3 s	32.7 s
	Fibrinogen	397 mg/dL	460 mg/dL	421 mg/dL
	D-Dimer	190 ng/mL	6671 ng/dL	378 ng/dL
	Antithrombin	101%	78%	85%
	Platelet	106000/mmc	41000/mmc	45000/mmc

	Coagulation parameter	Day of infusion	Day of the ADR	6 days after the infusion
		7/01/2020 (C1D1)	08/01/2020 (C1D2)	14/01/2020 (C1D8)
Patient 3	INR	0.98	1.18	0.97
	PT	10.3 s	12.4 s	10.2 s
	aPTT	29.9 s	34.3 s	29.5 s
	Fibrinogen	308 mg/dL	306 mg/dL	335 mg/dL
	D-Dimer	545 ng/dL	35200 ng/dL	637 ng/dL
	Antithrombin	91%	69%	101%
	Platelet	145000/mmc	23000/mmc	113000/mmc

	Coagulation parameter	Day of infusion	Day of the ADR	6 days after the infusion
		21/08/2018 (C1D1)	22/08/2018 (C1D2)	28/08/2018 (C1D8)
Patient 4	INR	1.09	1.31	1.08
	PT	11.7 s	14.2 s	11.7 s
	aPTT	—	35.7 s	32.9 s
	Fibrinogen	297 mg/dL	294 mg/dL	314 mg/dL
	D-Dimer	914 ng/dL	23690 ng/dL	948 ng/dL
	Antithrombin	85%	69%	83%
	Platelet	113000/mmc	82000/mmc	114000/mmc

* INR, international normalized ratio; PT, prothrombin time; aPTT, activated partial thromboplastin time; C, cycle; D, day. E.g., C1D1 = cycle 1, day 1.

The next step was an assessment of the medical literature evidence. We searched MEDLINE using English language as the only restriction. The Medical Subject Heading (MeSH) and the keywords used were: (“disseminated intravascular coagulation”) AND (“obinutuzumab”). No observational studies documented any possible association between obinutuzumab and DIC. Obinutuzumab-related non-overt DIC was described in only one case report (Walter et al., 2016) involving a 68-year-old woman with CLL treated with obinutuzumab and venetoclax. The patient had trisomy 12, a condition associated with an increased risk of IRR and the NOTCH1 mutation, which is associated with an unfavorable CLL prognosis. Trisomy 12 and NOTCH1 mutation have opposite effects on the expression of CD20: the CD20 expression was increased by trisomy 12 and decreased by NOTCH1. The coagulation parameters before the infusion were in the normal range. The patient developed an IRR 30 min after starting the infusion, so the infusion was halted, and the patient was treated with corticosteroids with adverse event resolution. Obinutuzumab infusion was restarted and completed without any other problems. However, upon completion of the infusion, the patient again developed alterations of the coagulation parameters: a grade 3 thrombocytopenia (almost 25 × 10^9^/L), prolonged prothrombin (18.4 s; normal range 12.0–15.0 s) and aPTT times (36.5 s; normal range 24.0–31.0 s), and a reduction in the fibrinogen level (1.5 g/L; normal range 2.0–4.0 g/L) and an increase in D-dimer (>20.00 μg/mL; normal range 0.0–0.5 μg/mL) were observed. Other coagulation pathway factors were decreased: factor II 46% (normal range 78.7–115.5), factor X 64% (normal range 73.1–132.7), and factor V 47% (normal range 53.8–127.7). The patient was treated with one unit of platelets for grade 3 thrombocytopenia. After 36 hours, the parameters turned normal except for thrombocytopenia, and the D-dimer level resolved, respectively, 1 week and 3 months later. Medical scientific literature was also searched for evidence supporting the hypothesis of a possible pharmacological mechanism explaining a causal role of obinutuzumab in the occurrence of non-overt DIC. Freeman et al. (2015) and Freeman et al. (2016) previously showed that obinutuzumab can induce the release of proinflammatory cytokines, such as interleukin-6 (IL6), IL-8, interferon γ (IFN-γ), and tumor necrosis factor (TNF). Interestingly, the peak of cytokines—IL-8 and IL-6 in particular—was observed during Day 1 of the first cycle and was followed by a rapid decrease, being the level of such cytokines close to 0 at Days 8 and 15. Furthermore, on Day 1 of the second cycle, the level of all cytokines was 0 except for IL-8, which was close to 0 and lower than that observed on Day 15 of the first cycle. It is well known that there is a mutual interplay between inflammation and coagulation. Tissue factors play a central role in activating coagulation during inflammation. Tissue factors can bind to factor VIIa, catalyzing the conversion of factor X to Xa and therefore increasing the production of thrombin (IIa) and the conversion of fibrinogen in fibrin. Furthermore, inflammation and thrombin itself stimulate platelet activation. Among the cytokines, IL-6 plays a major role in activating coagulation. In contrast, other cytokines such as TNF have been shown to play a pivotal role in the regulation of plasminogen activators and inhibitor activity. Cytokines may induce the release of both plasminogen activators (i.e., tissue-type plasminogen activator and urokinase-type plasminogen activator), causing plasmin generation, and plasminogen activator inhibitor type 1, which in turn inhibit fibrinolysis. Taken together, these two mechanisms are responsible for the concomitant risk of thrombosis associated with bleeding and hemorrhage (Freeman et al., 2015; Freeman et al., 2016). A possible class effect of anti-CD20 mAbs was also considered. A second assessment of the literature was carried out using these MeSH and key terms: (“disseminated intravascular coagulation”) AND (“anti-CD20” OR “rituximab” OR “ocrelizumab” OR “ibritumomab”).

At the time of analysis, rituximab, ocrelizumab, and yttrium-90 ibritumomab tiuxetan were the only three anti-CD20 mAbs approved for clinical use. In particular, ibritumomab tiuxetan [^90^Y] is a radiopharmaceutical preparation used also as a consolidation therapy. In the Italian PV database, there were two ICSRs for rituximab and DIC, while the publicly accessible EudraVigilance database (https://www.adrreports.eu/) recorded 170 ICSRs for DIC, in which rituximab was the suspected drug. Among the 170 cases of interest, 29 were characterized by a fatal outcome. No ICSRs were retrieved during the search for ocrelizumab and ibritumomab tiuxetan and DIC in both the national and European databases. Furthermore, DIC, although reported in the scientific literature for rituximab (Freeman et al., 2016; Rafei et al., 2017), was not labeled in the SPC of any of the medicinal products containing rituximab (European Medicines Agency, 2022b). DIC was also not labeled for ocrelizumab (European Medicines Agency, 2022c).

At the same time, AIFA checked the EudraVigilance database with the access level for national medicine regulatory authorities, which gives access to all data elements of ICSRs submitted to EudraVigilance for other ICSRs of obinutuzumab-induced DIC. Overall, seven supportive cases for obinutuzumab and DIC were retrieved. Among these, three cases were reported during clinical trials and the remaining four were the already known Italian cases. Interestingly all seven patients developed a non-overt DIC soon after the first dose of obinutuzumab. The DIC resolved spontaneously in all patients without any action needed for obinutuzumab treatment, including discontinuation. In light of all available evidence, the signal was then discussed and validated in April 2021 by the AIFA working group. The signal was then entered into EPITT (number 19711) in June 2021 and was confirmed by the PRAC Rapporteur (Sweden) in July 2021 (European Medicines Agency, 2021).

The signal moved further along the management path from “signal analysis and prioritization” and PRAC after the completion of the investigation, assessing and confirming DIC as a possible adverse reaction to obinutuzumab according to the majority of evaluated supporting cases represented by non-overt DIC. In February 2022, the following risk minimization measures were adopted: a special warning for DIC was included in the SPC at point 4.4 on the management of patients undergoing obinutuzumab administration. Paragraph 4.8 was also updated and the PT “disseminated intravascular coagulation” with “uncommon frequency” was inserted (European Medicines Agency, 2022d).

## Discussion

The signal detection process involves a sequence of several steps requiring the actual collaboration of the different stakeholders at the national level, triggering the expected activities of the EU PV system. These steps have been developed and standardized to produce a balanced assessment that aims to benefit the population in two ways: 1) protecting the patient from the actual and unknown risks of a drug and 2) protecting the drug (and the expected benefits) from false risks and unnecessary restrictions on its use without any proven motivation, depriving patients of a precious if not unique treatment tool. This evaluation process needs to be both efficient and rapid. However, both efficiency and speed depend on and are affected by various epidemiological and clinical factors; the case of obinutuzumab and DIC is quite paradigmatic for analyzing some of these factors.

The identification of DIC as a potential adverse reaction to obinutuzumab has required approximately 7 years after its marketing authorization. To trigger the signal and begin risk assessment, we had to wait for a rather long period of observation of treated patients—still necessary and unavoidable to collect sufficient data. This physiological latency of case identification or even the reporter was quite long due to the inherent difficulty in suspecting a causal link between obinutuzumab and DIC. Indeed, the presentation of DIC is frequently asymptomatic and can only be diagnosed by alterations in blood chemistry parameters. In these situations, the suspicion of a causal relationship is rarely triggered by a single case: the observer needs more cases with similar characteristics to report the event as an adverse drug reaction. Furthermore, underlying conditions, such as onco-hematological disease in the present case, could further mask the problem, representing a risk factor or an alternative cause.

Once the possible problem had been identified, the process of confirmation and implementation of risk minimization measures took about a year. In this case, identification could be affected and delayed by primarily epidemiological factors and limitations that are intrinsic to the disproportionality analysis (Noguchi et al., 2021). The rarer a reaction is, the more difficult it is to confirm in adequate observational studies. For obinutuzumab and non-overt DIC, there is no precise information on the frequency of the event due to diagnostic difficulties. This has also resulted in a very limited number of spontaneous reports, which usually help define the outcomes of a study aimed at confirming a signal. Difficulties related to the therapeutic setting could further increase the study design complexity and produce further slowdowns. The choice of an adequate comparator drug could be a problem in relation to the line of treatment or the presence of other drugs in the oncological treatment protocols. Even an observed/expected estimate implies the availability of information on the frequency of the DIC event in the reference population (adults with chronic lymphocytic leukemia) which, to the best of our knowledge, is not available in the literature. The clinical relevance of the event, on the other hand, can change the speed of the evaluation process, leading, for example, to the adoption of “provisional” risk minimization measures, such as, for example, a general recommendation of caution pending the results of an ongoing inquiry, and the invitation to collaborate by reporting similar events. For the present case, although the event was unexpected, the circumstance of the asymptomatic occurrence of DIC and its spontaneous resolution lowered the priority of the signal.

Analysis of the scientific medical literature is essential to consolidating the available evidence. For obinutuzumab-related DIC, the lack of evidence produced in clinical trials or observational studies was verified. The only clinical evidence available was related to a single case report. However, very important support for the confirmation of the signal derived from the identification of studies that allowed us to hypothesize a pharmacological mechanism linking obinutuzumab to DIC. When, as in this case, the evidence suggests that the mechanism of the adverse reaction may be linked to the mechanism of action that defines the therapeutic effect of the drug (collateral or side effect), it is always appropriate to verify a potential drug class effect. This verification was carried out by a comparison with the available evidence of a possible association with the other anti-CD20 agents used in therapy.

## Conclusion

In this study of the safety signal, we illustrated the process of identifying and confirming the DIC as a signal associated with obinutuzumab treatment for chronic lymphocytic leukemia. The path followed for the confirmation of the signal and the adoption of risk minimization measures by the Italian Medicines Agency within the framework of the EU procedures proved to be efficient and fast, considering the intrinsic difficulties specifically linked to the nature of the event itself. The study of the steps of the path and the standardization of the clinical investigation processes could further implement and strengthen the future efficiency of the system.

## Data Availability

The original contributions presented in the study are included in the article/Supplementary material; further inquiries can be directed to the corresponding author.
